# Enhanced Remediation of Polycyclic Aromatic Hydrocarbons in Soil Through Fungal Delignification Strategy and Organic Waste Amendment: A Review

**DOI:** 10.1007/s12088-024-01383-7

**Published:** 2024-09-09

**Authors:** Victor Taghoghor Omoni, Paul Olusegun Bankole, Kirk Taylor Semple, Adesola Samson Ojo, Cynthia Ibeto, Stephen Efe Okekporo, Idorenyin Ambrose Harrison

**Affiliations:** 1 Lancaster Environment Centre, Lancaster University, Lancaster, LA1 4YQ UK; 2 Department of Microbiology, College of Biosciences, Federal University of Agriculture, P.M.B. 2373, Makurdi, Benue State Nigeria; 3 Department of Pure and Applied Botany, College of Biosciences, Federal University of Agriculture, P.M.B. 2240, Abeokuta, Ogun State Nigeria; 4 Department of Pure and Industrial Chemistry, University of Nigeria, Nsukka, Nigeria; 5 Department of Botany, College of Biosciences, Federal University of Agriculture, P.M.B. 2373, Makurdi, Benue State Nigeria

**Keywords:** Organic materials, PAHs, Immobilisation, Biological pre-treatment, Fungi

## Abstract

Nutrient-limited soils from growing global contamination with polycyclic aromatic hydrocarbons (PAHs) and the massive organic waste generation from agro-based and food industries have raised more demand for exploring and recycling the latter as sustainable, cost-effective, and green nutrient-rich sources for soil amendment. To further enhanced the potentials of these substrates in soil, immobilisation or biological pre-treatment techniques using fungi are employed. The white-rot fungi- basidiomycetes, are the most widely researched and efficient organisms to perform these functions because of their high lignin-degrading ability for organic materials, such as corn cob, straws, spent brewery grains, sugarcane bagasse, etc. This review addresses the importance of organic amendment to enhance the biodegradation efficiency of PAH from contaminated soils and it also highlights various biological techniques for improving PAH biodegradation using organic waste materials and white-rot basidiomycetes. This review will also show a better understanding of the concepts of fungal immobilisation and pre-treatment for PAH degradation in soil and show their insights as feasible and optimise techniques for developing remedial strategies for contaminated soils.

## Introduction

Pollution from indiscriminate disposal of polycyclic aromatic hydrocarbons (PAHs) and other emerging contaminants exert negative impact on soil quality. These organic pollutants found their way into the soil through various channels, natural sources and anthropogenic activities such as bush/forest burning, exhaust fumes from cars, oil spillage and smoking. Organic pollutants get into the environment via biogenic, petrogenic and pyrogenic processes [1].

A major mechanism for the elimination of soil’s hydrophobic contaminants (HOC) is through microbial degradation [2]. However, the efficiency of HOC removal from the soil is largely dependent on the soil’s organic and mineral features, associated soil’s physical properties (soil texture, soil water, nutrient availability, and temperature) and physicochemical properties of the contaminants [2–5]. In addition, Omoni et al. [6] also emphasized the importance of availability of degrading microbes and bioavailable fractions of the HOC. Soils and sediments are natural sinks for HOCs in the environment via sorption processes [7, 8]. The mobility, degradation and bioavailability of HOC may affect their persistence in the environment over time [9]. Microorganisms play a critical role in the removal of contaminants in the environment through the application of microbes which is termed bioremediation. This technology is highly dependent on the vital role played by the microbes and enhanced induction of microbial enzymes that are capable of mineralizing the contaminants. However, a major criticism against bioremediation technology is the slow rate in the process of ameliorating the effect of contaminants in the environment, hence, a good strategy or tool is desirable in enhancing the removal process rate [6, 10, 11].

Biodegradation of HOC such as PAH underlies the bioremediation technology and its success relies on the interplay of forces between hydrocarbon degraders and the soil. Hence, the effective removal of PAH in the soil is largely influenced by the availability of organic carbon and nutrients [3, 12]. This fact was corroborated by Azubuike et al. [13] who reported that the efficiency of microbes to metabolize, mineralize, detoxify, degrade, and catabolize PAHs depends on the number of bioavailable nutrients [13]. In addition, the metabolic function of microflora and associated enzymes are reduced or remain inactive in soils such as nutrient-depleted soils, or those encountered with low contaminant availability, which can result in the persistence of an organic contaminant, and consequently, this can further affect the soil ecosystem’s health [14, 15]. The environmental consequences of nutrient-limited soils are well-known [16, 17]. For example, the high cost and complexities associated with the clean-up and reclamation of PAH-contaminated soils in most regions with oil contamination such as in Nigeria’s Niger-Delta zone [18–20]. Similarly, soil fertility and health, as well as microbial activities are drastically affected in such nutrient-depleted soils contaminated with petroleum hydrocarbons [21, 22].

Inherent nutrients present in agricultural wastes when applied to soil offer a sustainable strategy for ameliorating environmental challenges posed by PAH contamination in the environment. Specifically, lignocellulosic waste materials from agro-industrial processes such as tea waste, rice husks, bean husks, sugarcane bagasse, pea husks, groundnut cake wastes, spent brewery wastes, wood shavings, maize cobs have been used over the years to promote optimal growth and enhanced proliferation of the microbes and catabolism of soil contaminated with PAHs [11, 23–25]. Recently, there is a growing interest in augmenting organic waste material with biochar to further improve remediation in nutrient deficient soils [26] and PAH-contaminated soil [6, 27]. Augmenting and amending the soil with biochar have proven to enhance soil’s physicochemical properties whilst acting as enriched sources of organic macro and micro nutrients [28]. The use of organic wastes for soil amendments typically provides the soil with abundant carbon, nitrogen, and phosphorus for the growth, proliferation and survival of the soil’s micro and macro flora [29, 30]. More so, amendment of soil with biochar alters the microbial community structure and activities. Most of the agro-industrial wastes are produced in African and Asian regions of the world [11, 31]. In Africa, most especially, these wastes constitute a major menace to environmental pollution as a result of indiscriminate disposal of solid wastes and non-enforcement of existing waste disposal legislation [11].

Existentially, microbes make use of organic waste as ecological support for enhanced decontamination of soil. These wastes enhance the massive production of fungal mycelia, induction of ligninolytic enzymes and offer protection for both fungi and enzymes in unfavourable environmental conditions associated with HOC contamination [32–34]. Biotreatment of lignin-degraders (white-rot, soft-rot, brown rot fungi and certain bacteria strains) is another strategy used in processing organic waste for delignification to enhance organic pollutants biodegradation. However, the mechanism of pollutant removal lies in the rate of induction of extracellular enzymes by the rot fungi [35, 36]. The cellulose and hemicellulose delignification potentials of white-rot fungi have been reported widely by several workers [37, 38]. The fungal pre-treatment of organic waste is done to remove non-metabolizable lignin and expose the readily metabolizable carbon and nitrogen for microbial growth and metabolism.

Therefore, to further address the complexities, difficulties and high cost associated with PAH removal from soil a cost-effective, feasible, safer, sustainable and green approach to soil remediation are necessary. It is however important to review several techniques which can improve soil quality using organic waste materials for soil amendment. This is important for optimising bioremediation processes, maximising the benefits of the organic waste amendment and minimising the cost of treatment of PAHs in fresh and historically contaminated soils. In the present review, we present and discuss (i) an overview of organic waste materials and their importance in soils; (ii) organic waste materials and potentials in the bioremediation of PAHs in contaminated soil; (v) microbial mechanisms of PAHs and technologies available for removal of PAHs in soil; (v) role of white-rot fungi and fungal enzymes in organic waste materials in soils; (iv) an assessment on the use of fungal pre-treated and fungal immobilised on organic materials for the removal of PAHs in soil. The knowledge presented in this review may further ascertain that organic waste materials are still likely one of the silver bullets to soil restoration/reclamation with petroleum hydrocarbons (in terms of cost-effectiveness, feasibility, sustainability, safety, and practicality) or whether more investigation is still required.

## Organic Wastes and Amendments in Soil

Organic wastes are created mainly by industrial, agricultural, and household activities. These wastes are regarded as by-products or end-products of the production and consumption processes. Presently, most producers of organic wastes either pay huge amounts for their disposal or illegally dump them in the environment a practice common in Africa and parts of Asia alongside environmental, economic and social impacts. In most developed nations, large tonnes of organic wastes end up in landfills where the biodegradable materials generate and emit greenhouse gases. Currently, the reuse, recycle and recover approach for waste management, including energy recovery (waste-to-gas) has further improved its usability and sustainability for agriculture and other promising areas. The beneficial use of the waste materials is lost and unidentified. Alternatively, the organic waste materials can be used not only for waste-to-energy conversion but also as rich sources of microbial nutrients, enzymes and organic matter for soil amendments [39]. However, these wastes are highly diversified in nature and compositions and thus their formulation and application rates in soil should be considered.

Most developed and developing countries in the world generate a huge quantity of waste (estimated as 7–9 billion tonnes annually) from different industries [40] of which about 80% of the total wastes are organic [41]. To date, the vast majority of waste in the United Kingdom comes from the food production sector. Waste generation in Africa has reached an all-time high of about 62 million tonnes/year. The larger chunk of these wastes is of organic origin with an undocumented record in most member African nations. As a result, this has contributed negatively to the menace of environmental pollution bedevilling the African continent as there are no safe waste disposal strategies deployed in most communities [11]. In China, food worth over 200 billion Yuan is thrown away annually with an estimate of 40–90 million tons reported in 2015 [31].

In recent times, organic wastes have been composted to stabilise organic matter, reduce the moisture content, increase the concentrations of plant nutrients, reduce greenhouse emissions and eliminate pathogens [42, 43]. Also, there is increasing interest in processed organic wastes such as biochar as feedstock in organic waste composting because they can improve the composting process by enhancing organic matter decomposition, humification and microbial activities, as well as reduce nitrogen, greenhouse gas emissions, sequester heavy metal and organic contaminants [44]. Organic wastes can replace inorganic fertilisers in soil amendments and can be used as an alternative for large fertiliser applications in soil [45].

Positive effects of the application of organic wastes have been previously reported as a beneficial soil amendment in agriculture [39, 46] and in remediation practices [11, 24, 25, 42]. Organic amendments added to the soil can improve the physiochemical and biological qualities by altering soil pH, providing limiting nutrients and improving the water-holding capacity of the soil [47, 48]. Some organic wastes generated from different industrial processes, especially in the food industry such as spent brewery grains (SBG), spent and mushroom compost (SMC), biochars (processed organic wastes) have been gaining attention in recent years. Other organic waste materials with soil beneficial properties include straws, rice husk, rice straw, wheat straw, corn hub, sugarcane bagasse, animal manures, and wood chips. These materials can enhanced soil organic matter as well stimulate the soil biological activity [39, 41]. The use of organic waste materials for soil amendments is cleaner, cheaper, safer, feasible, and sustainable for nutrient application to soil.

### Spent Brewery Grains (SBG)

The beer industry is the fifth most consumed beverage in the world in both developed and developing countries apart from tea, carbonates, coffee and milk [49]. In the food industry, the brewery industry holds strategic economic importance with annual beer production exceeding 1.94 billion hL in 2018 [50] and 1.91 billion hL in 2019. It is estimated that approximately 40 million tons of SBG are produced globally which is expected to rise in the future with increasing beer production [51]. Of the global output, the European Union produced approximately 3.5 million tonnes of SBG annually. The brewery industry generates large amounts of these by-products (SBG) and wastes from their raw materials; spent grain, spent hops and yeast being the most common. The brewery industry is greener as most of the waste products can be recycled and reused for several beneficial purposes. The brewery industry uses several batch-type operations (successive separation) in processing the raw materials to the final beer product with about 85% of the total by-products containing SBG.

SBG is non-hazardous and biodegradable lignocellulosic waste with a high nutritive value which are composed of rich fibres (cellulose, hemicellulose and lignin) and protein which represent approximately 30–50% and 19–30% of its composition [52]. The percentage of cellulose, hemicellulose and lignin contents in SBG ranged from 18.4–37.2%, 13.8–26.8%, and 9.9–17.1%, respectively [53]. The lignin (a recalcitrant polyphenolic polymer) and protein contents are significantly high in SBG. Hemicellulose and cellulose are fractions constituted by sugars including glucose, xylose, and arabinose as the most abundant in SGB as well as traces of rhamnose and galactose [54]. It also contains trace lipids and other minerals such as silicon, phosphorus, calcium, iron, potassium, magnesium, manganese, sodium, cobalt, copper, and various vitamins [52, 55]. However, the chemical composition of SBG is a function of the barley variety used in beer production, the harvest time, mashing condition, the quality and types of adjuncts or the technology employed in the brewery processing [56].

As a result of their rich chemical composition, they have been useful in many fields, including agriculture, environment and energy, chemical and animal feeds. They are used as raw materials in biotechnological processes such as the productions of many valuable products [55]. Microorganisms (bacteria and fungi) identified in brewery wastes include *Pseudomonas* sp., *Acetobacter* sp., *Aspergillus niger*, *Curvularia* sp. and *Alternaria* sp. [57]. They are also used for the cultivation of microorganisms, enzyme production, and as carriers (pre-treatment and immobiliser) for both enzymes and microbes in the bioremediation of organic contaminants in soil [11, 58]. SBG has also been reported as absorbent material for heavy metals and the treatment of wastewater [54]. In hydrocarbons contaminated soils including PAHs, SBG have played key roles in soil amendment providing vital limiting nutrients for microbial growth and metabolism. Unfortunately, in some parts of the world, a large quantity of SBG goes to landfills promoting environmental degradation through carbon emissions, thereby adding to the carbon footprint.

### Compost

Compost is a source of organic matter and humus-rich for improving soil’s biological, chemical and physical properties. Compost triggers humification processes that form humus (highly complex substance). Humus is a large group of natural organic compounds found in the soil, formed from the chemical and biological decomposition of plant and animal residues as well as the synthetic activity of microorganisms [42]. These humus-like materials can be added to soil either through raw organic waste materials or matured compost produced by a composting process. Composting is not only an inexpensive natural process but also a more controlled process of aerobic biological breakdown of organic materials. Organic waste materials used as compost or in a composting process are usually green wastes. The green wastes (vegetable wastes, green clippings, food wastes, etc.) contain more nitrogen and less carbon compared to other agro-wastes including sawdust, corn straw, wheat straw, bark chips, agro-peels and which contain less nitrogen and more carbon which are more recalcitrant to microbial degradation owing to their constituents-cellulose, hemicellulose and lignin [59, 60]. Furthermore, green wastes contained large amounts of organic matter, they are associated with low environmental pollution and less investment is required [60]. However, nitrogen is more readily available in compostable materials than carbon due to the latter reaction with hydrocarbon compounds and becomes unavailable for microbial utilisation and also as carbon is bio-converted to CO_2_ during the process resulting in a lower C:N ratio. In composting production, C:N ratio is a key factor [61]. Most of the nitrogen in compostable materials is readily available. Some of the carbon, however, may be bound up in compounds that are highly resistant to biodegradation. For example, corn stalks and straws are difficult to break down because it consists of a resistant form of cellulose and lignin.

A large number of different genera of microorganisms are involved in composting process. Basically, composting is a dynamic process performed by a rapid succession of mixed microbial populations [62]. Compost consists of highly diverse and potentially active microorganisms [60]. Aerobic microorganisms are primarily involved in compost decomposition. Microbial communities’ dynamics and temperature changes are two characteristic successive features during composting which through a wide variety of metabolic processes under aerobic conditions forms mature compost (a humus-like material) [42]. Although the processes involved rapid sequential activity by a mixed variety of different microbial populations and their enzymes, for example, archaea, bacteria, fungi and actinomycetes decomposing the biomaterials [63] but this is dependent more on the starting material. In the early mesophilic stage, there is an increase in bacteria numbers due to high supply of easily degradable compounds present in the materials while the chemical dynamics and metabolic processes depend on the composition of the organic input material and optimal process conditions [42].

Composting process can be carried out on both small and large scales. However, a better technique determines the higher quality of the compost [62]. There are three composting techniques designed to accelerate and optimize the process on a large scale: windrow composting, static piles and closed vessel composting (In-vessel composting) [42, 62]. Composting or compost addition can act as a nutrient supplement, organic conditioner, pH adjustor and bulking agents (e.g., improving the pore size, gas space and water-holding capacity) in soil. However, the process is most feasible when all the physicochemical and biological factors are considered especially in soil bioremediation [42].

### Biochar

Biochar is an organic material enriched in carbon. It is usually obtained after pyrolytic treatment of biomass in anaerobic or microaerophilic conditions [64–66]. Biochar can be produced from wastes and by-products generated in forestry, agriculture, municipal waste, animal dungs, and plant remains [67] Depending on the intent of usage, biochars are produced in varying particle sizes, different heating temperatures, and times through the carbonisation process [68]. Temperature is the main determining factor affecting the characteristics of biochar. The solid material produced after pyrolytic transformation of the lignin and cellulose component of the wood is called Biochar. Gas, condensed tar, liquid fuel and charcoal are by-products usually obtained after pyrolytic treatment of wood shavings [69]. The wide applicability of biochar is due to the possession of unique characteristics such as stability, enlarged surface area, high porosity and cation exchange capacity. The ease of preparation, reusability, and economical and eco-friendly nature confers a favourable edge on biochar over other organic materials.

Biochar has many benefits in soils and can be used as a soil additive as well as the sequestration of carbon and improving soil properties in soil amendments, thus promoting sustainable agriculture [70]. Biochar has been produced from many agricultural waste materials including rice straw, wheat straw, sawdust, waste wood, sugar beet tailings, corn cob, etc. for soil amendments [68, 71]. It is a source of direct and valuable mineral nutrients such as N, P, Ca and K because it is produced from pyrolyzed rich biomass and can also improve soil nutrients bioavailability.

Biochar can be mixed with other inorganic nutrients to improve soil productivity. Alburquerque et al. [72] reported about a 20–30% increase in the yield of wheat grains in soil amended with both wheat straw biochar and fertiliser than fertiliser alone. The effects of biochar addition to soil depend on its characteristics, production and feedstocks, soil and crop types and land management [73]. When in soil, biochar can exhibit positive or negative effects. Several studies have reported the positive effect of biochar application on the soil. For example, biochar can influence soil microbial growth, abundance and activity [6, 74], chemical alteration [75], improve water holding capacity and saturated conductivity owing to a large number of tiny pore sizes [76]. Some of the negative effects reported include a reduction in cation exchange capacity (CEC), no change in pH in temperate soil as well direct risk to soil biota and their functions [77]. In addition, biochar produced at high temperatures can persist in soil for a longer time. This however depends primarily on the soil and biochar type.

The interaction of biochar with the soil usually leads to the formation of bifunctional groups. The groups offer more active sites on the biochar thus enhancing its actions to retain organic compounds. The oxidation process may result in; an increased amount of oxygen and hydrogen and decrease the carbon content of the biochar; the formation of functional groups containing oxygen; and decreasing the negative charge of the biochar surface [78]. This interaction ability of biochar is largely enhanced by the temperature used during pyrolysis and the addition of oxidative chemicals [78]. The ageing of biochar in soil can however increase the negative charges in biochar through further oxidation, thus influencing its biochemistry in soil. The particle size of biochar also influences the soil quality and stability, attack, degradation rates and mineralisation by soil biota. For example, Thie and Rillig et al. [79] reported increased microbial colonisation due to increases in surface area of smaller particle size biochar after soil amendment. Additionally, biochar with larger surface area promotes microbial activities and contaminant binding sites and sorption for heavy metals, pesticides, and PAHs [71].

Biochar has been utilized in curbing many environmental issues such as adsorbing pollutants such as heavy metals, reducing greenhouse emissions, composting, wastewater treatment, soil remediation, and energy production [80]. Biochar is gaining more attention from many researchers in establishing its efficiency in the removal of various contaminants. The potential of biochar in adsorbing/remediating organic and inorganic pollutants depends on the high surface/volume ratio and its affinity towards nonpolar groups [68]. The addition of biochar to soils improves the growth, survival and activity of soil microbial community by increasing the pore fraction of soil which in turn enhances the moisture, air, and residence time of microbes in pore fractions [68].

## Lignocellulosic Waste Materials in Soil Bioremediation with PAHs: Sustainability and Environmental Clean-Up.

Lignocellulosic wastes have been deployed over the years as metabolic support in the eco-friendly, and economical strategy in the bioremediation of petroleum hydrocarbon contaminated environment [81]. A variety of biological and physicochemical reactions are stimulated when lignocellulosic wastes are applied to soil [82, 83]. As a result of soil amendment with organic wastes, microbial activities are significantly enhanced (Table 1). Amendments made with lignocellulose act through two mechanisms, namely adsorption and mineralization [84].

**Table 1:** Chemical composition of different lignocellulosic materials that can be utilised for biological pre-treatment and immobilisation [53, 185–188]

Lignocellulosic by-products	Cellulose (%)	Hemicellulose (%)	Lignin (%)
Banana waste	13.2	14.8	14
Barley straw	31–34	24–29	14–15
Bast fibre jute	45–53	18–21	21–26
Buffalo manure	0.4	6.0	7.4
Coffee pulp	35	46.3	18.8
Corn cobs	35–45	35–45	5–15
Coir pith	29	15	31
Cotton waste	80–95	5–20	NA
Flax straw	29	27	22
Fruit and vegetable wastes	7.2–43.6	4.3–33.5	15.3–69.4
Groundnut shell	36	19	30
Hardwood barks/chips	22–40	20–38	30–55
Pine	46	24	27
Poplar wood	35	17	26
Primary wastewater solids	8–15	NA	24–29
Qat straw	31–37	27–38	16–19
Rye straw	33–35	27–30	16–19
Rice straw	25–35	20–30	10–15
Wheat straw	29–35	26–32	16–21
Saw dust	28–34	17–21	25–32
Softwood barks/chips	45–50	25–35	25–35
Solid cattle manure	1.6–4.7	1.4–3.3	2.7–5.7
Sorted refuse	60	20	20
Spent mushroom compost	5.3–27.0	36.5–51.9	24–36.8
Sewage sludge compost	55.3	41	270.8
Spruce	47	22	29
Spent brewery grains	18.4–37.2	13.8–26.8	9.9–17.1
Sugarcane bagasse	32–44	25–35	19–24
Sweet sorghum bagasse	45	25	18
Swine wastes	6	28	NA
Wastepaper from chemical pulps	60–70	10–20	5–10

Researchers have examined organic wastes as soil conditioners during PAH remediation processes involving catabolism and enzyme induction. Further, soil management practices such as adding organic and inorganic amendments may influence soil community composition, structure, and microbial biomass [83, 85]. Table 2 shows how PAHs are removed from soil amended with organic waste materials (lignocellulosic biomass).

**Table 2:** Lignocellulosic waste used in soil amendment during PAH biodegradation from contaminated soil

Lignocellulosic by-products	PAH	Percentage degradation/removal	References
Corn cobs	15 EPA PAHs	16% (Total PAHs), 16% (benzo[a]pyrene), 44% (anthracene), decreasing order of % degradation: 3-ring > 4-ring > 5- and 6-ring	[189]
Rice straw	2–6 rings PAHs	30.3%	[190]
	Anthracene, pyrene, benzo[a]pyrene	> 96% (anthracene and pyrene), > 52–60% (benzo[a]pyrene)	[191]
	Phenanthrene	64%	[192]
Wheat straw	13 PAHs	dibenz[a,h]anthracene (52%), anthracene (46%), Total 13 PAHs (23%)	[25]
Wheat stalk	16 PAHs	LMW (59.3%), HMW (24.3%), Total PAHs (33.3%)	[89]
Saw dust	2–6 rings PAHs	66.3%	[190]
Spent mushroom compost	Phenanthrene	35%	[11]
	Napthalene, phenanthrene, benzo[a]pyrene, benzo[ghi]perylene	Napthalene (84%), phenanthrene (59%), benzo[a]anthracene, (68%), benzo[ghi]perylene (68%)	[193]
	Phenanthrene	42.7%	[194]
	16 PAHs	LMW (60.2%), HMW (31.2%), Total PAHs (38.7%)	[89]
Sewage sludge compost	Anthracene, pyrene, benzo[a]pyrene	> 96% (anthracene and pyrene), > 52–60% (benzo[a]pyrene)	[191]
Spent brewery grains	Phenanthrene	48.7%	[11]
Fruit and vegetable wastes	16 EPA PAHs	49%	[97]
Food and Kitchen waste	16 EPA PAHs	56%	[97]
Buffalo manure	16 EPA PAHs	49%	[97]
Pinewood biochar	13 PAHs	Dibenz [a, h] anthracene (59%), fluorene (34%), Total 13 PAHs (14%)	[25]
Enhanced biochar	Phenanthrene	45.6%	[6]
Non-enhanced biochar	Phenanthrene	32.6%	[11]

Macro-and micronutrients supplied by organic materials are capable of stimulating active and relevant microbial activity and positively influencing microbial structure and diversity, thereby increasing microbial degradation of organic contaminants in soil [6, 11, 86]. In the literatures, organic materials have been demonstrated to improve soil physicochemical and biological properties [25, 87]. In addition, these biomaterials can act as bulking agents and microbial biomass suppliers within the soil, increasing the levels of soluble carbon, nitrogen, and phosphorus as well as reducing soil sorptive sites and enhancing microbial uptake and degradation of contaminants [81]. According to Gaind et al. [88], soil amended with organic materials enriched with P has higher biological activity than soil amended with inorganic materials. In nutrient-depleted soils, the use of organic waste materials as soil amendments has therefore contributed to improved environmental sustainability and performance [89].

### Soil Amendment with SBGs

Microbial biomass, nitrogen, phosphorus, organic solids, and microbial biomass can be found in brewery wastes (grains and effluents). Although, the potential of SBG for soil remediation has been studied very rarely. However, Omoni et al. [11] and Abioye et al. [90] reported that SBG could stimulate proliferation of enzymes by indigenous fungi needed for effective remediation of PAH-contaminated soil. Abioye et al. [90] specifically reported that nutrient elements, biomass and biosolids in soils were exponentially enhanced after biostimulation of pollution impacted soil with SBGs. As a result of SBG addition, indigenous biota can be stimulated to bioremediate PAH-contaminated soils [11, 90, 91].

According to Abioye et al. [90], biostimulation of contaminated soils laced with lubrication oil with amendments from SBG, banana peels and SMC significantly improved PAH removal rates by 68.73, 62.03, and 57.01%. Abioye and his co-workers further reported that PAH removal efficiency from soil amended with SBG was more practicable than the performance exuded by other co-substrates. The biodegradation kinetics of phenanthrene in amended soil was altered by SBG application with increasing mineralization rates of 10% and 20%, according to Omoni et al. [11]. Over a 100-day period of soil-PAH interactions, enhanced proliferation of bacteria and fungi were also observed in amended soils. PAH remediation should not include large amounts of organic materials as soil additives. In soil amended with SBG, PAH degradation has only been reported in this study. An appropriate amount of SBG application should also be considered in contaminated soils, since high organic matter in soil can reduce the bioavailability of PAHs to PAH-degraders, reducing rates of mineralisation and thus influencing the extent of mineralisation [11].

### Soil Amendments with Compost

A sustainable technology is desirable for the rejuvenation of pollution-impacted soils where reduced chemical toxicity before biodegradation is generally necessary. Nonetheless, organic wastes can be used for bioremediation in hydrocarbon-polluted soil. Organic substrates have shown promising potential as adsorbents for bacterial and fungal attachments, subject to their biodegradability. By enhancing microbial nutrients with organic substrates, compost-mediated bioremediation is highly regarded as the economical, eco-friendly, and super-enrichment technique for the remediation of pollution impacted environment [92] and as a choice to chemical co-substrates. In contaminated soil-compost mixtures, stable compost (compost stability) derived from organic wastes (biosolids) and balanced soil-waste amendments promote the biodegradation of PAH contaminants [93–95]. When added to contaminated soil, immature or unstable compost can decrease microbial activity and metabolism. In general, compost adds organic matter to the soil, which not only increases microbial activity but also lowers the bioavailability of contaminants in low concentrations [42].

The addition of compost to PAH-impacted soil usually leads to a surge in bound residue thus stimulating microbial activity and removal efficiency [96, 97]. The formation of pollutant-humic acid complexes in the soil is reduced when organic matter is humified. While it is evident that humic complexes in compost sometimes act as PAH carriers, increasing their solubility, bioavailability, and desorption to microbial cells, while posing negligible toxicity to soil [98, 99]. A recent study found that soil amended with fresh compost had significantly lower PAH levels and was less accessible to microorganisms that degrade PAHs [98]. A higher percentage of PAHs may be sequestered to soil organic matter when soil matrices have a high PAH concentration above acceptable or background levels, which can negatively affect degradation. By adding compost to such soils, it can modify and balance organic sorption and biodegradation, thus improving soil quality.

Compost has both physical and chemical properties that can aid soil biota in recolonizing niches of the compost microbes and in desorbing contaminants from the soil that are present that have been contaminated [42]. When lignocellulosic is included in the composting process, lignin-degrading fungal colonizers are encouraged to produce exoenzymes, which can help to degrade HOCs. SMC derived from *Pleurotus ostreatus*, *Lentinus edodes*, or *Agaricus bisporus* can be used as additives and extracellular-degrading enzymes for recalcitrant organic pollutants including PAHs, dyes, and pharmaceuticals [42, 100].

Composting processes result in the development of highly complex metabolites and the inactivation of microbes involved. This acts as a super-augmentation for biological stimulation, thus leading to high metabolic activities in the soil during remediation [42]. As shown in Table 2, PAHs biodegrade efficiently in SMC amended soils.

### Soil Amendments with Biochar

It has been proven that biochar (also known as pyrolyzed agricultural residues) binds and biodegrades soil’s persistent organic pollutants. For biochar degradation, two mechanisms have been proposed: surface oxidation and microbial degradation [101]. The use of biochars can improve soil properties in terms of physicochemical aspects as well as biological aspects [26, 102], as well as help, biodegrade PAHs in soil [85, 89, 103, 104].

The absorption capacity of biochar makes it an excellent source of micro- and macronutrients in soil [105]. Freshly prepared biochar releases N amounts ranging from 23 to 635 mg kg^−1^ and P amounts ranging from 46 to 1664 mg kg^−1^ [106]. Agro-residues and various forms of biochar have been linked to the biogeochemical cycling of nutrients as a result of their addition to soil produced from pyrolyzed wastes. After the amendment, sewage sludge biochar can increase soil microbial activity by supplying adequate amounts of micronutrients and macronutrients [107].

PAH absorption onto biochar has proven to be very effective in reducing toxicity and associated environmental risks [108–110]. According to Kusmierz et al. [111], PAHs were not only persistent in acidic soil but also increased in soil content; however, 3- and 4-ring PAHs were lost rapidly from soil amended with biochar. As shown in Table 2, some pyrolyzed organic waste materials are efficient at removing PAHs. Singh and Cowie [112] claim that biochar stimulates native SOC mineralization in a low-C clayey soil, but the effect diminishes over time, possibly due to the depletion of labile SOC from initial positive priming, and/or the stabilisation of SOC induced by biochar. In PAH-contaminated soils, biochar can also stimulate enzyme activities [104, 113]. Moreno Jiménez et al. [110], found that biochar properties (feedstock, pH, micronutrients, and soil properties) may differ. Based on Brzostek et al. [114], SOC stimulated ligninolytic enzymes, acid phosphatase, and phenol oxidase. There is evidence that soil amended with biochar reduces the amount of substrates such as OM like mineralization and sorption, and this affects some key enzyme production in soil [115].

Zhang et al. [26] conducted studies on the biodegradation of naphthalene, phenanthrene, pyrene, and chrysene in amended soils containing walnut shells, corn cob, corn stems, and rice straw at three different temperatures of 250°, 400°, and 600 °C. The biochar made from walnut shells at 400 °C degraded pyrene (40%) and chrysene (60%) at a higher rate than biochar made from corn stems (84 and 50%, respectively). Additionally, the researchers found that chrysene degraded faster in biochar-treated soils than pyrene, presumably because it was more accessible to the biochar due to its hydrophobicity (log *Kow*) compared to pyrene. Furthermore, in contaminated soils, biochar may support and preserve soil biodiversity and biotopes for micro and mesobiota, thereby supporting the preservation and restoration of soil biodiversity and biotopes [116].

## Other Organic Materials in Soil Amendments

Biodegradation of PAHs has been observed in soil amended with nutrient-enriched organic substrates from household and agricultural origin (Table 1). It has also been shown that soil amended with organic substrates reduces the negative effects of PAHs on soil microbes’ interactions and enzyme induction. Aryl alcohol oxidase, glyoxal oxidase dehydrogenase, and beta-glucosidase are stimulated in soil by their presence. These organic materials contribute to massive environmental pollution, making their management and disposal increasingly challenging. Bioenergy residues called anaerobic digestate have recently been reported as rich nutrients and soil conditioners by microbial communities anaerobic digesting biodegradable wastes and sewage sludge without oxygen in controlled environments [117]. Anthracene, phenanthrene, pyrene and fluoranthene were amended to marine sediment with digestate, fresh organic fraction of solid municipal waste, and combinations of micro- and macronutrients, resulting in 55% increased degradation efficiency compared to the control (12%). Specifically, Ibeto et al. [118] reported that when anaerobic digestate fractions were amended to phenanthrene-contaminated soils, the kinetics of biodegradation and soil physiochemistry are enhanced. Thus, they conclude that it is necessary to ensure the biosafety of this organic nutrient before it can be used as a fertilizer in agriculture.

### Role of Lignin-Degrading Fungi in Lignocellulose Degradation

#### White-rot fungi (*Basidiomycetes*)

**Fig. 1:**
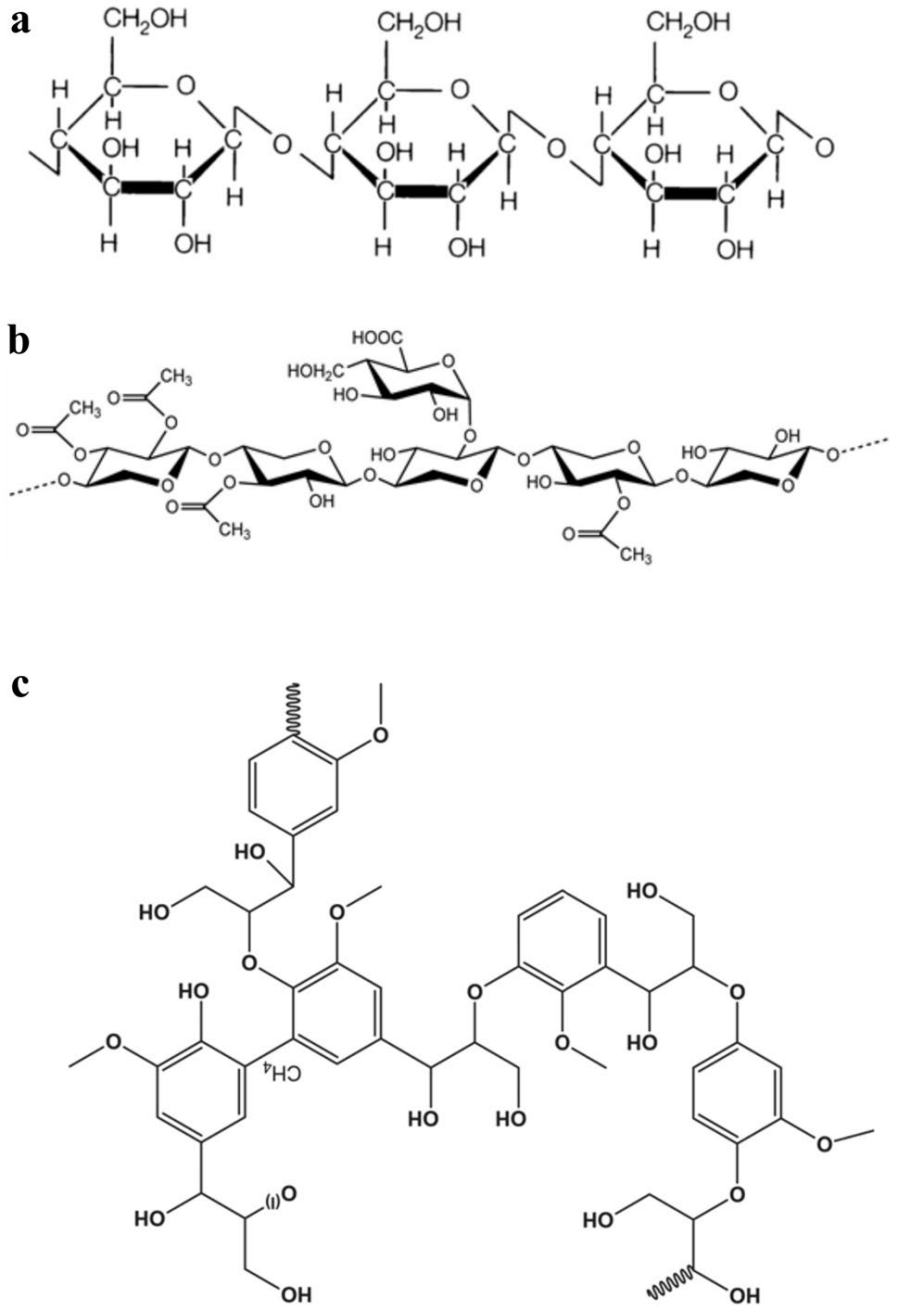
**a** Molecular structures of **a** cellulose [182], **b** hemicellulose [183] and **c** lignin, comprising the three basic phenylpropanoid monomers (aromatic ancestors)- *p*-hydroxyphenyl (H), guaiacyl (G), and syringyl (S) units, linked from three aromatic precursors *p*-coumaryl, coniferyl, and sinapyl, respectively [184]

Over 90% of wood-rotting fungi are white-rot fungi (WRF), which are a heterogeneous group of *Basidiomycetes* fungi [119]. Compared to other lignocellulose degraders, WRFs are capable of degrading all the components (cellulose, hemicellulose, and lignin). Figure 1 shows the chemical structures of cellulose, hemicellulose, and lignin. After wood degradation, a white residue or bleached form is left behind. Sharma and Arora [120] noted that these fungi produce lignin-modifying enzymes (laccase, lignin peroxidase and manganese peroxidase), hemicelluloses and cellulases. The WRF also possesses both extracellular and intracellular cytochrome P-450 monooxygenase-epoxide hydrolase, which synergizes with ligninolytic enzymes to degrade organic contaminants [121, 122]. Each eukaryotic organism contains the cytochrome P-450 epoxide hydrolase enzyme. Secondary metabolism involves the secretion of ligninolytic enzymes by WRF. Some WRFs such as *Phanerochaete chrysosporium* genes encoding lignin-modifying enzymes are known to be regulated by nutrient limitations (nitrogen, carbon, carbohydrate), oxygen, and metal ions (trace, Mg^2+^, Ca^2+^) in culture media [123], while ligninolytic enzymes produced by *Bjerkandera* sp. and *Pleurotus* species can be improved in N-sufficient conditions [124, 125]. Some of the WRFs with potential for selective delignification of lignocellulose include *Trametes versicolor*, *Pleurotus eryngii*, *Pleurotus ostreatus*, *Pleurotus pulmonarius,* *Phanerochaete* *chrysosporium,* *Stropharia* *coroilla,* *Dichomitus squalens*, *Coriolus versicolor*, *Pycnopus cinnabarinus*, *Ceriporiopsis subvermispora* and *Cyathus stercoreus*. The white-rot fungi such as *Phlebia* spp., *C*. *subvermispora*, *Phellinus* *pini*, *Phellinus* *pini* and *Pleurotus* spp. can delignify wood by preferentially attacking lignin over hemicellulose and cellulose, leaving enriched cellulose. Several white-rot fungi degrade plant cell walls simultaneously, including *Irpex lateus*, *Trametes versicolor*, and *Heterobasidion annosum* [126].

#### Brown-rot fungi (*Basidiomycetes*)

These groups of fungi belong to basidiomycetes and can only slightly modify lignin while rapidly metabolising cellulose and hemicellulose. More than 7% of all wood-rotting fungi are of the brown-rot type. Hence, they are not considered to be lignin degraders. Also, they have no ligninolytic enzymes except for small molecule reactive species to depolymerise lignin [127]. Brown-rotted wood or residual lignin residue is dry, brownish (as a result of high concentration of modified or oxidised lignin), excessive shrinkage with decreased strength and typically degraded into cubical fragments [128]. All brown-rot fungi belong to basidiomycota such as *Gloeophyllum trabeum*, *Fomitopsis pinocola*, *Fomitopisis lilaccinogilva*, *Rhodonia* (*Postia*) *placenta*, *Laetiporus portentosus*. They modify lignin through non-enzymatic degradation compared to white-rot fungi. Brown-rot fungi modify lignocellulose by an oxidative radical-based system using iron-dependent Fenton chemistry known as chelator-mediated Fenton system (CMF). The CMF system is a unique substrate deconstruction system based on oxygen radical chemistry that allows non-enzymatic rearrangement (depolymerisation, repolymerisation and demethylation) of the cellulose structure [127, 129]. While both brown-rot and white-rot fungi can reduce pH levels in their immediate environment, the brown-rot is associated with more significant pH reductions [130, 131]. Additionally, brown and white rot fungi produce oxalate that plays an indirect role in lignin degradation and pH gradient development [131].

#### Soft-rot fungi (*Ascomycetes *and Anamorphic Fungi)

Soft-rot decay of wood is caused by certain species of *Ascomycetous* and mitosporic fungi [132]. The degradation results in the darkening and softening of the wood, and the residue is cracked when dry. These characteristics are associated with the ligninolytic enzymes involved in the modifications [127]. But these enzymes are unspecific and limited in function compared to those secreted by brown-rot and white-rot fungi. Cellulase enzyme produced by soft-rot fungi can break down cellulose leading to the formation of microscopic cavities inside the wood, and they are found in dry environments with high moisture, low aeration, high temperature and the presence of preservatives which are inhibited by the other wood-rotting fungi [36]. Soft-rot fungi belonging to the genera such as *Daldinia*, *Hypoxylon* and *Xylaria* caused erosion soft rot. There is little knowledge of the mechanisms involved in the degradation of lignocellulose by these rot fungi.

#### Ligninolytic Enzymes and Non-Ligninolytic (Auxiliary) Enzymes

Approximately two major groups of enzymes have been identified for lignin degradation, namely the lignin-degrading auxiliary enzymes (also called accessory enzymes) and the lignin-modifying enzymes. In order to fully degrade lignin, additional enzymes are required to degrade lignin [123]. Several proteins, including oxidative H_2_O_2_, are involved in the sequential action of lignin-degrading auxiliary enzymes [123]. A number of such enzymes belong to this group, such as cellobiose dehydrogenase, glyoxal oxidase, aryl alcohol oxidases, glucose oxidase, and pyranose 2-oxidase [123, 133]. For lignin degradation, various enzymes are involved, including lignin peroxidases, manganese peroxidases, and versatile peroxidases. Lignin polymers containing phenolic and nonphenolic groups are depolymerized by ligninolytic enzymes via free radicals of lower molecular weight (hydroxyls), and the insoluble lignin is mineralized [134, 135]. There is a well-documented group of ligninolytic enzymes found in white-rot fungi [127]. The white-rot group of fungi, in particular, may utilize lignin-containing biomass as a source of carbon to produce chitin, carbon dioxide, and water [133].

Laccase (EC 1.10.3.2) is one of the polyphenol oxidases often referred to as blue multicopper enzymes capable of oxidising a variety of phenolic and non-phenolic compounds [136, 137]. Phenolic compounds, including lignin, polyphenols, methoxy-substituted phenols, or diamines, are oxidised by one-electron oxidation that can lead to formation of repolymerise or depolymerise radicals [127]. Based on the Cu atoms dispersion among the three altered binding sites and its function, laccase enzyme is divided into three groups, namely ring cleavage, of organic compounds, (2) degradation of biopolymers and (3) cross-linking structure of monomers [123]. Lacasse active site contains three copper sites: type 1 (T1, one CU atom), type 2 (T2, one Cu atom), and type 3 (T3, two copper atoms) per molecule of laccase [127]. During the oxidation by laccase, the T1 site accepts electrons from reducing substrates, and these electrons are transferred internally from T1Cu site onto the trinuclear T2 and T3 clusters where the molecular oxygen is activated and reduced to water [123, 138]. Laccase secretion is mostly favoured in acidic to near neutral pH and it is produced by the basidiomycetes and ascomycetes; however, laccase is mostly produced by white-rot fungi. In the presence of a redox mediator, 2,2′-azinobis- 3-ethylbenzthiazoline-6-sulfonate (ABTS), that behaves as an efficient electron shuttle from the substrate of the primary electron donors to the electron-accepting compounds [139]. Laccases have been found at high levels and studied in lignin-degrading fungi, such as *Trametes versicolor*, *Trametes pubescens*, *Trametes hirsuta*, *Coriolus hirsutus*, *Pycnoporus cinnabarinus*, *Pleurotus ostreatus* [127]. Some other fungi species such as *Lentinus tigrinus*, *Coniophora puteana*, *Neurospora crassa*, *Pycnoporus sanguineus* and *Chaetomium thermophile* [140] are also able to produce laccases.

Lignin peroxidases (EC.1.11.14) are lignin-modifying enzymes that belong to the heme-containing peroxidases and are known as peroxides oxidoreductases, oxidising nonphenolic methoxyl-substituted lignin units in the presence of H_2_O_2_ [127]. These enzymes were first discovered in cultures of *Phanerochaete* c*hrysosporium* [126]. Lignin peroxidase works in an acidic environment and its isozymes are glycoproteins that have a molecular weight (MW) of 40–68 kDa with four carbohydrates, 370 water molecules, 343 amino acids residues, two calcium ions, and a heme group [123]. LiP isozymes have been identified in *Phanerochaete* c*hrysosporium Phanerochaete sordida*, *Trametes versicolor* and *Phlebia radiata* [141]. During LiP oxidation, a two-step reaction is employed which involves the native enzyme in its ferric resting state, the radical cation oxoferryl intermediate compound II [142]. Additionally, LiP enzymes can oxidize substrates by electron transfer through a multistep process [126]. As a result of one electron abstraction, these enzymes are capable of oxidizing various aromatic organic compounds with redox potentials higher than 1.4 V. However, the redox mechanism that takes place is not fully understood [123]. It is also characterized by the ability to oxidize a wide range of aromatic compounds, hence, its role in the enzymatic degradation of lignin. Apart from the characteristic oxidation of non‐phenolic substrates, LiP has also shown the ability to oxidize a variety of phenolic compounds [123]. LiPs are produced by basidiomycetes and only in a few white-rot fungi.

MnP (EC 1.11.1.13) has been described as the most common lignin-modifying peroxidase secreted by most white-rot fungi and litter decomposers [141]. White-rot fungi secrete MnP in multiple forms such as the 11 different MnP isoforms secreted by *Ceriporiopsis subvermispora* [143]. It is the most important enzyme in lignin degradation. These enzymes belong to the heme-containing enzyme belonging to the oxidoreductase family [123]. MPs are produced by lignocellulosic fungi in both solid and liquid media in the microenvironment. The MnP enzymes are found in basidiomycetes and the enzymes are common in white-rot fungi and some other lignin-degrading fungi [144]. Both the structure and mechanism of LiP and MnP are similar in *P*. *chrysosporium* [126]. The mechanism of action of MnP includes the catalytic oxidation of Mn^2+^ to Mn^3+^, which is highly reactive and can diffuse into the lignified cell wall, where it oxidizes a wide range of phenolic substrates including the lignin phenolic structures and nonphenolic components [145]. However, MnP also possesses the ability to oxidize or cleave non-phenolic derivative compounds with the contributions of a second mediator, including thiyl or lipid radicals [123]. Wood-decaying fungi that are high producers of MnPs include *P*. *chrysosporium*, *Trametes* spp., *Phlebia* sp., *Physisporinus* r*ivulosus*, and *Pleurotus* spp. [146–148].

#### Lignin-Degrading Fungi and Ligninolytic Enzymes in Soil Bioremediation

**Fig. 2:**
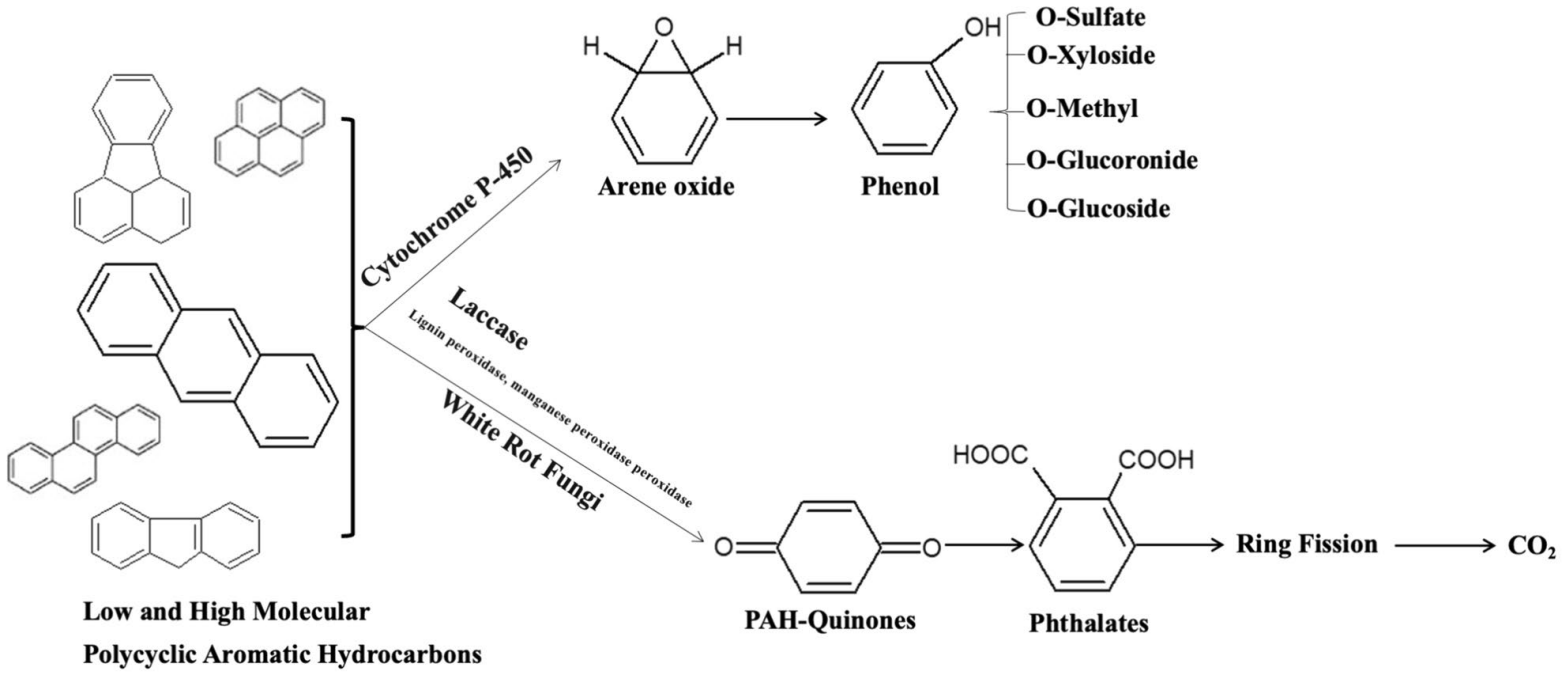
Metabolic pathways of degradation by fungi

For fungi to degrade PAHs in contaminated sites, carbon sources such as corn cobs, straw, sawdust, and sugarcane bagasse (Table 1) are important resources for energy. Additionally, fungi can colonize contaminated soil more efficiently with their branching filamentous growth mode. Compared to bacterial systems, fungi do not require preconditioning to the particular pollutant. For bacteria to produce enzymes that degrade a pollutant, they must be prior exposure to the pollutant. Furthermore, bacteria can only degrade pollutants up to a finite level if they are in significant concentrations; otherwise, enzyme synthesis cannot be induced [149]. The WRF is one of the lignin-degrading fungi extensively studied for its ability to degrade and mineralise PAHs, including those in *Trametes*, *Bjerkandera*, *Phanerochaete*, *Irpex*, and *Pleurotus,* and in the hyphomycete *Penicillium* and *Aspergillus* [121, 150–154]. However, the degradative function of each strain can differ depending on its ligninolytic enzyme complex [151, 154]. These groups of fungi secrete and/or stimulate ligninolytic enzymes most often in the presence of lignocellulose (Table 3). In addition to delignifying lignin-containing substrates through radical reactions, lignin-modifying enzymes are also capable of oxidating a range of organic compounds with similar structures, such as soil humic substances and organic contaminants [144, 155]. There has been previously reported involvement of intracellular cytochrome P-450 and epoxide hydrolase systems in the initial step in the degradation pathways of PAHs by WRFs [121, 156] as well (Fig. 2). Anthracene, fluorene, phenanthrene, and pyrene were all significantly degraded by *Rhodococcus *sp.,* Trichoderma tomentosum,* and *Fusarium oxysporum* in a study performed by Marchand et al. [157]. Anthracene, phenanthrene, fluorene, and pyrene were effectively removed by fungi species after 49 days, respectively [157]. Under saline-alkaline stress conditions, the white-rot fungus *Bjerkandera adusta* SM46 was also able to biotransform HMW benzo[a]pyrene when salinity increased to 20 g per liter. A study by Emuh [158] found that heavy metals and crude oil present in contaminated soil are absorbed into hyphae and mycelia of mushrooms through enzyme secretion, resulting in an increase in carbon dioxide (IV), water, and biomass production. According to Zebulun et al. [159], a white-rot fungus (*Pleurotus ostreatus*) enhanced biodegradation of anthracene-contaminated soil. Compared to control soil (35–31%), incubation time, contamination level, and fungal treatment significantly influenced anthracene degradation rates (76–89%). In polluted soil, *P*. *ostreatus* releases ligninolytic enzymes such as LiP, Lac, and MnP that contribute to the degradation of anthracene. PAH biodegradation is also affected by co-contaminants and mediators. Bhattacharya et al. [160] also found that 2,2′-azinobis-(3-ethylbenzothiazoline-6-sulfonate) and vanillin were important mediators in the degradation of benzo[a]pyrene by *Pleurotus ostreatus*. According to this study, 15 mM copper (Cu) was found to be the best enhancer of benzo[a]pyrene degradation (74.2%). By adding 5 mM of vanillin to the medium, the extent of degradation increased to 83.6%. Further, 150 different white-rot fungi species were evaluated in Korea for their performance on dye decolorization, gallic acid reaction, ligninolytic enzymes, and tolerance to four toxic PAH compounds: phenanthrene, anthracene, fluoranthene, and pyrene. In both individual and mixed PAH tests, six isolates showed the highest tolerance (> 90%). Lee et al. [161] found that *Peniophora incarnata* KUC8836 and *Phlebia brevispora* KUC9033 were able to degrade PAHs significantly in environmental settings.

**Table 3:** Degradation of PAHs in lignocellulosic waste amended soils

Rot fungus	Lignocellulose support	Treatment techniques	Detected ligninolytics	PAH	Time (days)	PAH dissipation/efficiency	References
*Trametes versicolor*, *Bjerkandera adusta*, *Phanerochaete chrysosporium*, *Pleurotus ostreatus and Irpex lateus*	Spent brewery grains	Immobilisation	Laccase, LiP and MnP	Phenanthrene	100 d	Immobilised fungi removed between 53.8–61.2% of 14C-phenanthrene in amended soils at 25 d. Immobilised *P. ostreatus* (61.2%) showed the greatest extent of degradation. *B. adusta* and *P. chrysosporium* also showed greater extents of mineralisation in most soil-PAH contact points	[165]
*Trametes versicolor*, *Bjerkandera adusta*, *Phanerochaete chrysosporium*, *Pleurotus ostreatus and Irpex lateus*	Spent brewery grains	Biological pre-treatment	Laccase, LiP and MnP	Phenanthrene	100 d	The extents of degradation were higher after 1 d (55.2 – 69.7%) incubation. Soil amended with *T*. *versicolor* pretreated SBG showed the highest removal (69.7%)	[164]
*Trametes versicolor*	Wheat straw/Wheat braw	Biological pre-treatment	Lacasse and MnP	4–5 rings	60 d	Generally, the extents of 14C-phenanthrene mineralisation in amended soils followed this order (*T*. *versicolor* > *B*. *adusta* > *P*. *ostreatus* > *P. chrysosporium* > *I*. *lateus*)*.* Fluoranthene and pyrene showed higher degradation rates ranging from 63 to 68% and 59 to 83%, respectively	[195]
*Lentinus trigrinus*	Wheat straw/Wheat braw	Biological pre-treatment	Lacasse and MnP	4–5 rings	60 d	As compared to fluoranthene, pyrene exhibited a higher degree of degradation, ranging from 63–60% and 59–83%, respectively	[195]
*Pleurotus ostreatus*	Mushroom cultivation substrate	Biological pre-treatment	Not measured	15 EPA PAHs	60-d	Among the 15 PAHs studied, 32.9% dissipated in amended microcosms. The most degradable PAHs are anthracene, pyrene and anthracene with > 60% removal observed	[196]
	Chopped wheat braw	Immobilisation	Not measured	13 PAHs	60 d	It is most effective at degrading the 3-ring PAHs (fluorene, phenanthrene, and anthracene), and moderately effective at degrading the 4-ring PAHs (fluoranthene and pyrene). The degradation rates of the members with 5 rings (benzo[a]anthracene, chrysene, benzo[b]fluoranthene, benzo[k]fluoranthene, and benzo[a]pyrene) varied between 32.8 and 85.2%	[197]
	Wheat straw	Immobilisation	LiP, MnP	13 PAHs	42 d	A total of 73% of the 13 PAHs were degraded. Following 21 days of incubation, dibenz[a,h]anthracene (75%) and fluorene (70%) showed the greatest degradation, while 94% and 81% of dibenz[a,h]anthracene and benzo[a]pyrene were removed after 42 days	[25]
*Phanerochaete chrysosporium*	Sugarcane bagasse	Biological pre-treatment	Not measured	Benzo(a)pyrene	5 d and 10 d	After 5 days of incubation, 68% of the benzo(a)pyrene was removed	[198]
	Sugarcane bagasse	Immobilisation	MnP	Anthracene	10 d	A 7-day shaken culture removed 84% of anthracene, while a free cell only removed 54%	[166]
	Pine sawdust	Biological pre-treatment	Not measured	Benzo(a)pyrene	5 d and 10 d	After 10 days of incubation, PS removed 74% of PAH	[198]
*Phanerochaete velutina*	Composted green wastes	Biological pre-treatment	LAC, MnP	16 EPA PAHs	30 d	Immobilised fungi degraded 96% of 4-ring PAHs and 39% of 5- and 6-ringed PAHs	[134]
*Bjerkandera adusta*	Rice straw	Immobilisation	LAC, LiP, MnP	Naphthalene, phenanthrene, chrysene, benzo(a)pyrene	30 d	Degradation of LWM-PAHs was greatest: naphthalene (94%), phenanthrene (70%) while chrysene (55%), benzo[a]pyrene (63%) of HMW-PAHs were degraded after 30 d. Mean degradation in the order of naphthalene > phenanthrene > benzo(a)pyrene > chrysene	[33]
*Dichomitus squalens*	Chopped wheat braw	Immobilisation	Not measured	13 PAHs	60 d	Phenanthrene and anthracene were degraded to 17.1% and 61.2%, respectively	[197]
	Ground corncobs	Immobilisation	Not measured	13 PAHs	60 d	The degradation of anthracene and phenanthrene was 15.9% and 68.1%, respectively	[197]
	Commercial pellets	Immobilisation	Not measured	13 PAHs	60 d	Anthracene and phenanthrene were degraded by 53.9% and 74.9%, respectively	[197]
*Penicillium frequentans*	Sugarcane bagasse	Biological pre-treatment	Not measured	Phenanthrene	17 d	With 20% oxygen, phenanthrene was removed in 52% after 17 days	[199]

#### PAH Biodegradation Through Biotreatment with Lignocellulosic Substrates and Immobilisation

The bioremediation of HOCs, including PAHs, can be achieved using immobilized fungi and catalytic enzymatic (ligninolytic) systems (intracellular and extracellular) [123]. This bioremediation strategy is cost-effective and efficient. Immobilisation of mycelia biomass protects the mycelial cells from the shear force, increasing cell/enzyme survival during storage, maintaining activity, and maintaining stability over different pH, temperatures, and incubation periods as well as providing a more suitable environment for the fungal cells and its enzymes [162]. The cells and enzymes are also more tolerant to toxic pollution, and the environment, such as heavy metals, is enhanced [34, 163].

**Fig. 3:**
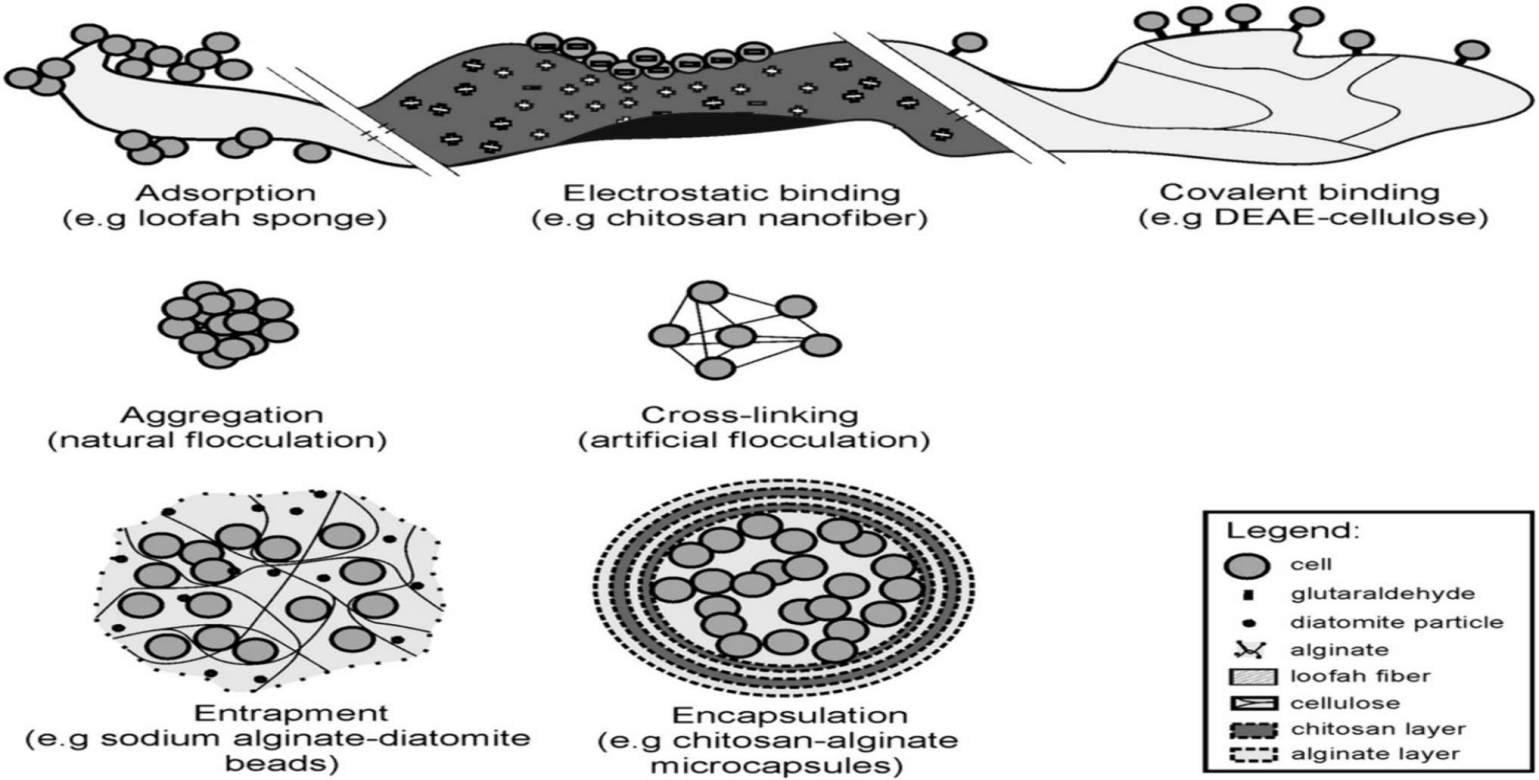
Immobilisation techniques [34]

The purpose of immobilization is to limit the mobility of microbial cells or enzymes while preserving their stability, viability, and catalytic activity [34] Fig. 3. There are several types of solid support materials that can be used to immobilize fungi or their enzymes, such as lignocellulose-containing material shown in Table 1 and Table 3. These co-substrates are biodegradable, water-loving, and economical with high surface porosity [34]. In this method, microorganisms formed biofilms on surfaces as well as using their inherent ability to attach to the surfaces. In the process of remediating contaminated soils, lignocellulosic substrates have been shown to induce a ligninolytic enzyme to degrade different organic compounds [123, 152, 164, 165]. Lignocellulosic materials are most effectively immobilized and pre-treated with white-rot fungi. Fungal immobilization of lignocellulosic materials and pretreatment of lignocellulosic materials have been explored for biodegradation of various organic compounds in the environment [165–167]. An overview of the lignocellulosic materials used for PAH mineralisation and the ligninolytic substances released by amended soils is provided in Table 3. This solid-state fermentation (SSF) boosts enzyme activity by stimulating the growth of fungi [164, 168].

The structure of lignocellulosic biomass residues and their chemical structures can be altered via several pre-treatment methods [169]. As opposed to mechanical, chemical, or thermal pretreatment methods that require high energy inputs and produced toxic inhibitory products, such as acetic, furical, and phenolic acids, biological pretreatment has some advantages. Additionally, chemical pre-treatment is expensive because of the high cost of the solvent used [170]. As a consequence, biological pretreatment processes are more economically viable, more effective, and more eco-friendly than other pretreatment processes [37, 171]. Biochemical pretreatments offer promising approaches for removing lignin from waste materials and producing monosaccharides (glucose, and galactose) that can be metabolized effortlessly by microorganisms. Furthermore, lignocellulosic materials are not the only solid carriers in bioremediation because natural and synthetic co-substrates are also deployed. As a result of the immobilization techniques used for bioremediation, these carriers can be classified into five different types: entrapment, adsorption, encapsulation, natural and artificial flocculation (natural and artificial) (Fig. 3).

Adsorption of microbial cells and enzymes to surface membranes such as lignocellulose materials is the easiest and commonest method used in bioremediation processes owing to its cost-effectiveness, rapid process, simple application, and greener strategy.

## Future Perspectives

Addition of organic materials, fungi immobilised and fungal-pre-treated organic materials, when amended to soil, have been proven to enhance the biodegradation of PAHs in contaminated soils. These waste materials and the delignification techniques (immobilisation and pre-treatment) with ligninolytic fungi, are effective for increasing the biological activities in soil. Microorganisms still remain the most important agents determining the fate of PAHs as well as the bioremediation of pollutants in the environment. Although microbial role may be hindered by poor nutrients, and abiotic factors, sometimes referred to as environmental factors such as soil and PAH properties. These factors will primarily influence the behaviour of microorganisms and their metabolic capabilities for PAH degradation in contaminated soil. Soil pH can determine the optimal biodegradation of PAHs; pH values within 6.5–7.5 favour most microbial activity and function. Most natural environments (soil and groundwater) possess often near neutral or slightly alkaline pH range with high buffering capacity, which is suitable for microbial growth, proliferation and degradation of environmental contaminants. pH values are decreased significantly with increasing soil-phenanthrene contact time when fungi immobilized and fungal-pre-treated on spent brewery grains were added. The reasons behind the reduction in soil pH and the metabolites produced should be examined before and after the amendments [12, 172] to identify the metabolites responsible. The buffer solution may be added to the soil to resist changes in soil pH before PAH degradation to support this process.

The use of organic materials in soil amendments, notably spent brewery grains, showed high potential to degrade PAHs and provide nutrients to boost soil biology. However, it is unknown what components of spent brewery grains cause these positive effects. Several organic pollutants, including phenanthrene, benzo[a]pyrene, phenol, and pentachlorophenol, were examined as part of Sun et al.’s [173] study. Despite its smaller polarity and increased aromaticity, pyrene exhibited the greatest sorption on lignin in this study. As well as a higher sorption coefficient than cellulose or hemicellulose, it also displayed a higher water absorption coefficient. A greater inhibitory effect was observed on cellulose and hemicellulose when phenanthrene was present than when lignin was present. Despite the sufficient sorption sites on lignin, competitive sorption was not as important as it once was. Phenol increased pyrene’s sorption to rice straw, lignin, and acetate, but inhibited the sorption on cellulose and hemicellulose. The presence of no aromatic compounds in pyrene, phenanthrene, and naphthalene made them more soluble in lignin than chitins and celluloses, according to Wang et al. [174]. Therefore, they conclude that soil bioavailability of PAHs is affected by the presence of lignin. A more detailed investigation of the biodegradation of PAHs in soil could be obtained by examining each purified constituent individually, as well as in combination with spent brewery grains.

By immobilizing lignocellulosic and PAH-degrading enzymes in soil, PAHs can be degraded more efficiently [122, 151, 175]. However, this strategy may be limited in its effectiveness when applied to hydrocarbon-contaminated matrixes such as those encountered during bioremediation in the field. The effectiveness of these materials in promoting PAH biodegradation will need further research. Additionally, the research was conducted under semi-controlled conditions, but to determine how organic wastes (spent brewery grains) and fungal treatment techniques can be used to treat and remediate hydrocarbon-contaminated soils, studies must be conducted in the field.

Mushroom compost and biochar applied at certain rates to soils have been shown to increase PAH mineralization [6, 11]. It is evident that a tripod mixture of mushroom compost, biochar and brewery grains could contribute to enhance degradation of PAHs in soil synergistically. Although further research is needed to investigate how these organic materials can be combined to enhance PAH biodegradation.

By increasing soil-PAH contact time may reduce the mineralisation of PAHs and biological activities in soil; thus, greater degradation and increased soil bioactivities can be achieved by increasing the bioavailability of PAHs with surfactant which in turn reduce the interfacial tension and improve its water solubility in the soil [176–178]. More so, the enzymes tend to decrease activity following increases in soil-PAH interaction indicating that the fungal secretion of enzymes was affected by decreased bioavailability, thus the use of surfactant may increase the enzyme secretion and stimulation.

It is recommended to investigate white-rot fungi with high degradative capacity for both PAHs and lignocellulose in hydrocarbon-contaminated soil following amendment. In addition, soil after lignocellulosic amendment should be assessed for the activity of accessory enzymes that do not degrade lignin, like aryl alcohol oxidases, glyoxal oxidases, cellobiose dehydrogenases, and superoxide dismutases [133]. Furthermore, adding organic and inorganic co-contaminants is needed to scale up treatment methods for displaced PAHs [179–181]. As a result, the contaminant will be more readily bioaccessible and mobile for microbial uptake and metabolism.

## Conclusion

Through the use of organic waste and white-rot fungi, PAH-contaminated soils can be remedied in an eco-friendly manner. The bioaugmentation of soils contaminated with PAH not only helps with remediation processes but also improves the soil’s nutrient composition. However, developing this green technology for field-scale applications requires the painstaking effort of optimization and feasibility studies. A study on microbial mechanisms should also be carried out to determine the underlying microbial factors that are responsible for the clean-up of the environment. In summary, organic amendment and white-rot fungi have proven to be effective, eco-friendly, and efficient methods for remediating PAH-contaminated environments.
